# Diabetes and liver cirrhosis: shared pathways and clinical implications – a narrative review

**DOI:** 10.1097/MS9.0000000000004012

**Published:** 2025-10-07

**Authors:** Anmol Mohan, Zim Warda Hasan, Hetvi Gordhan Galani, Tahniat Afroze, Rukash Khan Niazi, Razan Alfaki, Atika Shahzadi, Priyanka Mohan Lal, Hasibullah Aminpoor, Hasiba Karimi, Vikash Kumar, Usha Tejwaney, Sarwan Kumar

**Affiliations:** a Department of Internal Medicine, Carle Foundation Hospital, Urbana, IL, USA; b Clinical and Translational Science, School of Medicine, West Virginia University, Morgantown, WV, USA; c Department of Internal Medicine, Bukovinian State Medical University, Chernivtsi, Ukraine; d Department of Internal Medicine, Dr. VRK Women’s Medical College, Hyderabad, India; e Department of Internal Medicine, Sargodha Medical College, Sargodha, Pakistan; f Department of Internal Medicine, University of Khartoum, Khartoum, Sudan; g Department of Internal Medicine, CMH Kharian Medical College, Kharian, Pakistan; h Department of Internal Medicine, Ziauddin University, Karachi, Pakistan; i Faculty of Medicine, Kabul University of Medical Sciences, Kabul, Afghanistan; j Faculty of Medicine, Bezmialem Vakif University, Istanbul, Turkey; k Department of Internal Medicine, The Brooklyn Hospital Center, New York, USA; l Department of internal medicine, Valley Health System, Ridgewood, New Jersey, USA; m Department of Internal Medicine, Wayne State University, Detroit, Michigan, USA

**Keywords:** insulin resistance, liver cirrhosis, metabolic dysfunction-associated steatotic liver disease (MASLD), non-alcoholic fatty liver disease (NAFLD), type 2 diabetes mellitus (T2DM)

## Abstract

The global rise in type 2 diabetes mellitus (T2DM) and metabolic dysfunction-associated steatotic liver disease (MASLD) represents a critical and intertwined public health challenge. Both conditions share overlapping mechanisms, such as insulin resistance, lipid toxicity, chronic inflammation, and mitochondrial dysfunction, that drive disease progression and worsen outcomes. This review synthesizes emerging evidence on the bidirectional relationship between T2DM and MASLD, with a specific focus on underrecognized clinical intersections, including hepatogenous diabetes and the role of subclinical liver injury in diabetic patients. Unlike prior reviews, it emphasizes mechanistic links involving advanced glycation end products, cytokine dysregulation, and microvascular dysfunction, highlighting their implications for both hepatic and extrahepatic complications. We summarize epidemiological trends, outline gaps in current screening practices, and assess therapeutic strategies ranging from glycemic control and hepatoprotective antidiabetic agents to liver-directed interventions. Key takeaways for clinicians include the importance of early, integrated liver fibrosis assessment in diabetic care and the need for multidisciplinary approaches to reduce risks of cirrhosis, hepatocellular carcinoma, and mortality. By bridging pathophysiological insights with practical screening and treatment considerations, this review aims to guide more proactive and coordinated management of patients affected by T2DM and MASLD.

## Introduction

The global incidence of type 2 diabetes mellitus (T2DM) and metabolic dysfunction-associated steatotic liver disease (MASLD) is rapidly increasing, with shared pathogenic mechanisms linking these conditions^[1,2]^. Insulin resistance (IR) plays a central role in their pathophysiology, driving ectopic fat deposition in the liver and contributing to cardiovascular disease risk^[3]^. The association between T2DM and MASLD is bidirectional, with each condition influencing the development and progression of the other^[2]^. Common pathways include lipid toxicity, impaired autophagy, mitochondrial dysfunction, and chronic inflammation^[4]^. The co-existence of T2DM and MASLD increases the risk of adverse liver-related and extrahepatic outcomes, necessitating a multidisciplinary approach to management^[1]^. Effective strategies for screening, risk stratification, and co-management of these interrelated conditions are crucial for addressing this growing public health concern^[1,2]^.

**HIGHLIGHTS**

Our study:

- Examines shared mechanisms such as insulin resistance, chronic inflammation, and cytokine dysregulation that link type 2 diabetes mellitus and liver cirrhosis.
- Highlights clinical implications, including increased risks of hepatocellular carcinoma, hepatogenous diabetes, and diagnostic challenges.
- Proposes integrated screening and management strategies to support multidisciplinary care and improve patient outcomes.

The objective of this research is to explore the correlations between the rising prevalence, shared mechanisms, and clinical outcomes of T2DM and MASLD. Specifically, the paper aims to examine how IR, lipid toxicity, chronic inflammation, and other common pathogenic factors contribute to the bidirectional relationship between these two conditions, with an emphasis on their impact on liver-related and extrahepatic complications. By understanding these correlations, the study seeks to highlight the importance of early diagnosis, effective screening strategies, and integrated management approaches to improve patient outcomes and address the growing public health burden posed by both diseases.

The structure of the paper is designed to systematically address the topic, starting with an introduction that outlines the background and research objectives. It then provides an overview of the pathophysiology of both T2DM and cirrhosis, followed by a detailed exploration of the mechanistic links between the two. Epidemiological evidence of their correlation is examined, and clinical outcomes are discussed in the context of diabetes patients with liver dysfunction. The paper concludes with an exploration of screening, therapeutic considerations, and future research needs, emphasizing the need for integrated care to manage these interconnected conditions effectively. We confirm that no AI tools were utilized in the conduct or writing of this manuscript, in accordance with the TITAN checklist.^[5]^

## Methods

This narrative review was conducted to synthesize and critically appraise current evidence on the bidirectional relationship between T2DM and MASLD, with a focus on shared pathophysiological pathways and clinical correlations, including hepatogenous diabetes (HD). The methodology followed a structured but non-systematic literature review framework, allowing for broad coverage of mechanistic, epidemiological, and clinical aspects of the topic. A comprehensive literature search was performed between March and July 2024 across PubMed, Scopus, Web of Science, and Google Scholar databases. Keywords and MeSH terms included: “type 2 diabetes mellitus,” “T2DM,” “diabetes,” “metabolic dysfunction–associated steatotic liver disease,” “MASLD,” “non-alcoholic fatty liver disease,” “NAFLD,” “liver cirrhosis,” “hepatogenous diabetes,” “insulin resistance,” “lipotoxicity,” “inflammation,” “mitochondrial dysfunction,” “microvascular dysfunction,” “advanced glycation end products,” “fibrosis,” “hepatocellular carcinoma,” “mortality,” “screening,” “management.” Boolean operators (“AND,” “OR”) were used to refine results. Additional references were identified by manual screening of the bibliographies of relevant articles and recent reviews. Peer-reviewed articles, meta-analyses, systematic reviews, narrative reviews, clinical guidelines, and key mechanistic studies published in English from January 2000 to July 2024 were considered. Both human and relevant animal studies were included if they provided mechanistic insight or clinical correlation. Conference abstracts, non-peer-reviewed content, and studies without relevance to the T2DM–MASLD/cirrhosis relationship were excluded.

## Overview of both conditions

### Diabetes mellitus

T2DM is characterized by IR and relative insulin secretion deficiency, leading to chronic hyperglycemia^[6]^. The pathophysiology involves defective insulin secretion by pancreatic β-cells and impaired insulin sensitivity in target tissues^[7]^. IR, a key feature of T2DM, results from various factors including obesity, sedentary lifestyle, and genetic predisposition^[8]^. The disease progression involves a complex interplay of mechanisms affecting insulin synthesis, release, and sensing^[7]^. Environmental factors such as diet, physical inactivity, and gut dysbiosis contribute to T2DM development^[7]^. The pathogenesis is marked by a gradual decline in β-cell function and increased IR over time^[9]^. Understanding these pathophysiological mechanisms is crucial for developing effective treatment strategies, including pharmacological interventions targeting IR and β-cell dysfunction^[8]^.

### Cirrhosis

Chronic liver disease progression involves persistent inflammation, fibrosis, and hepatocellular injury, ultimately leading to cirrhosis, portal hypertension, and liver failure^[10,11]^. Inflammasomes play a crucial role in driving liver injury and inflammation, contributing to fibrosis and systemic effects^[10]^. Hepatic myofibroblasts are key players in fibrogenesis, resulting in excessive extracellular matrix accumulation^[12]^. Common complications of chronic liver failure include variceal bleeding, coagulopathy, hepatic encephalopathy, and renal failure^[11]^. Traditional staging systems based on histopathological assessment of fibrosis have limitations in representing the complexity of disease progression, particularly in cirrhosis^[13]^. The integration of invasive and non-invasive methods, along with consideration of etiology-driven mechanisms, is crucial for developing more accurate predictive indexes and improving clinical management of chronic liver diseases^[13]^.

## Mechanistic correlation between diabetes and cirrhosis

### IR and hepatic steatosis

IR plays a crucial role in the development of hepatic steatosis and non-alcoholic fatty liver disease (NAFLD). In IR, impaired insulin-mediated suppression of gluconeogenesis coexists with increased de novo lipogenesis (DNL) in the liver^[14]^. This leads to hepatic lipid accumulation through multiple mechanisms, including enhanced free fatty acid release from adipose tissue, increased DNL, decreased β-oxidation, and reduced very low-density lipoprotein (VLDL) secretion^[15,16]^. The “first hit” in the multiple-hit model of liver injury is IR and associated metabolic disturbances, which make the liver vulnerable to subsequent hits such as oxidative stress, dysregulated apoptosis, and pro-fibrogenic pathways^[17]^. Understanding these molecular mechanisms is crucial for developing targeted therapies for NAFLD and associated metabolic disorders^[15,16]^.

### Progression to NASH and cirrhosis

NAFLD can progress to non-alcoholic steatohepatitis (NASH) and cirrhosis through chronic inflammation, lipotoxicity, and hepatocyte ballooning^[18]^. Lipotoxicity, caused by the accumulation of toxic lipids like free fatty acids and cholesterol, triggers hepatocyte death, inflammation, and fibrosis^[19,20]^. Diabetes accelerates NAFLD progression, with diabetic patients at higher risk for advanced disease^[21]^. The pathogenesis involves multiple organs, including dysfunctional adipose tissue releasing toxic lipids and alterations in the gut–liver axis contributing to inflammation and fibrogenesis^[18,19]^. Excess free cholesterol in hepatocytes, Kupffer cells, and hepatic stellate cells plays a critical role in NASH development through proinflammatory and profibrotic effects^[20]^. Understanding these mechanisms is crucial for developing effective prevention strategies and therapies for NASH patients^[19]^.

### Proinflammatory and profibrotic cytokines

Proinflammatory cytokines play a crucial role in liver fibrosis and diabetes pathogenesis. In hepatic stellate cells (HSCs), cytokines like IL-1β and TNF-α can reverse the pro-fibrogenic phenotype, increasing matrix metalloproteinases and inflammatory markers^[22]^. However, TGF-β1 promotes fibrosis by downregulating metalloproteinases and upregulating collagen production^[22]^. In NASH, TNF-α and other proinflammatory cytokines modulate the necro-inflammatory component, while factors regulating TGF-β dictate fibrosis progression^[23]^. Diabetes is now considered a chronic inflammatory disease, with IR associated with increased TNF-α and IL-6 production^[24]^. In type 1 diabetes, Th17 and Th22 responses, along with TGF-beta and STAT3 upregulation, are involved in the immune response^[24]^. Understanding these cytokine interactions is crucial for developing targeted therapies for liver fibrosis and diabetes.

### Advanced glycation end products

Advanced glycation end products (AGEs) play a significant role in liver fibrosis and NAFLD progression. AGEs exacerbate chronic liver injury and contribute to hepatic fibrosis^[25]^. In HSCs, AGEs induce reactive oxygen species (ROS) production through NADPH oxidase activation, potentially promoting liver fibrosis in metabolic syndrome^[26]^. The interaction between AGEs and their receptors in HSCs increases ROS production, enhancing cell proliferation and activation, which worsens liver fibrosis^[27]^. Furthermore, toxic AGEs suppress apoptosis in activated HSCs and increase collagen I production, potentially aggravating liver fibrosis in chronic hepatitis conditions like NASH^[28]^. These findings highlight the complex interplay between AGEs, oxidative stress, and liver fibrosis, suggesting potential targets for therapeutic interventions in NAFLD and related liver diseases.

### Microvascular dysfunction

Microvascular dysfunction is a key feature of diabetes mellitus, leading to complications such as retinopathy, nephropathy, and neuropathy^[29,30]^. This dysfunction precedes structural changes in the vasculature and can serve as an early marker for diabetes^[30]^. The pathogenesis involves hyperglycemia, increased microvascular pressure and flow, and endothelial injury^[31,32]^. Oxidative stress, hyperosmolar stress, and inflammatory pathways also contribute to microvascular impairment^[32]^. Different patterns of microvascular abnormalities are observed in insulin-dependent and non-insulin-dependent diabetes^[31]^. Early detection of microvascular dysfunction is possible through various technologies and computational methods^[30]^. Management strategies include lifestyle modifications, particularly exercise programs, which can slow the progression of microvascular dysfunction and its associated complications^[30]^. Understanding these mechanisms provides opportunities for developing novel preventive and therapeutic interventions^[29,32]^. Overview of these mechanisms correlating diabetets and cirrhosis are summarized in Table 1.

**Table 1:** Mechanistic correlation between diabetes and cirrhosis

Mechanism	Description
Insulin resistance (IR) and hepatic steatosis	- IR impairs suppression of gluconeogenesis and enhances de novo lipogenesis.
	- Leads to hepatic lipid accumulation via increased FFA release, reduced β-oxidation, and decreased VLDL secretion.
	- Forms the “first hit” in liver injury, sensitizing the liver to oxidative stress and fibrogenic pathways.
Progression to NASH and cirrhosis	- NAFLD may progress to NASH and cirrhosis due to chronic inflammation and lipotoxicity.
	- Lipotoxicity triggers hepatocyte death, inflammation, and fibrosis.
	- Diabetics are more prone to NASH and cirrhosis.
	- Dysregulated adipose tissue and gut–liver axis contribute to inflammation and fibrosis.
	- Excess cholesterol in liver cells drives proinflammatory and profibrotic responses.
Proinflammatory and profibrotic cytokines	- IL-1β, TNF-α can alter the phenotype of hepatic stellate cells (HSCs), promoting fibrosis.
	- TGF-β1 increases collagen and reduces matrix degradation, contributing to fibrosis.
	- Diabetes is associated with increased TNF-α, IL-6, and Th17/Th22 responses.
	- Chronic inflammation is a shared hallmark of both diabetes and liver disease.
Advanced glycation end products (AGEs)	- AGEs promote ROS production and HSC activation via NADPH oxidase.
	- Lead to increased collagen production and cell proliferation.
	- Toxic AGEs (TAGEs) suppress apoptosis and promote fibrosis, particularly in NASH.
	- AGE–TAGE interaction plays a critical role in liver fibrosis in metabolic disorders.
Microvascular dysfunction	- A key diabetes complication, affecting multiple organs.
	- Driven by hyperglycemia, increased vascular pressure, endothelial injury, oxidative and osmolar stress.
	- Seen early in diabetes and varies by diabetes type.
	- Early detection possible with computational methods.
	- Lifestyle modifications, especially exercise, can delay progression.

NAFLD, non-alcoholic fatty liver disease; NASH, non-alcoholic steatohepatitis; ROS, reactive oxygen species; FFA, free fatty acids; VLDL, very low-density lipoprotein; NADPH, nicotinamide adenine dinucleotide phosphate (reduced form); TGF, transforming growth factor; IL, interleukin; TNF, tumor necrosis factor.

## Epidemiological evidence of correlation

### Prevalence studies

NAFLD) and its more severe form, NASH, are highly prevalent among patients with type 2 diabetes (T2D). Studies have shown that the prevalence of NAFLD in T2D patients can be as high as 70%, with NASH affecting approximately 37% of diabetics^[33]^. Even in T2D patients with normal aminotransferase levels, the prevalence of NAFLD was found to be 50%^[34]^. NAFLD is more common in obese and Hispanic individuals, with diabetes being a significant risk factor^[35]^. The prevalence of NASH in diabetics (18.5%) is higher than in non-diabetics (7.1%)^[36]^. T2D patients with NAFLD face an increased risk of disease progression to cirrhosis, hepatocellular carcinoma (HCC), and mortality^[33]^. These findings emphasize the importance of screening T2D patients for NAFLD/NASH, even when liver enzymes appear normal.

### Longitudinal data

Diabetes mellitus (DM) has been identified as an independent risk factor for liver cirrhosis and HCC progression. Studies show that DM patients have a two- to threefold higher risk of developing cirrhosis^[37]^. DM is associated with various liver diseases, including NAFLD, alcoholic cirrhosis, and chronic hepatitis C^[38]^. A meta-analysis of cohort studies revealed that DM significantly increases the risk of HCC incidence (SRR = 2.01) and mortality (SRR = 1.56), independent of geographic location, alcohol consumption, cirrhosis history, or viral hepatitis infections^[39]^. Furthermore, diabetic patients with alcoholic liver disease or NAFLD have a higher probability of developing cirrhosis (60% vs. 41%) and HCC (27% vs. 10%) compared to non-diabetics over a 3-year follow-up period^[40]^. These findings underscore the importance of monitoring liver health in diabetic patients.

### Bidirectional risk

Chronic liver diseases, particularly cirrhosis, are strongly associated with glucose intolerance and HD. About 80% of cirrhotic patients exhibit glucose intolerance, with 20–30% developing diabetes^[41]^. HD prevalence in cirrhosis ranges from 30–60%^[38]^. The pathophysiology involves IR in muscle, liver, and adipose tissues, along with hyperinsulinemia and impaired pancreatic β-cell response^[38,41]^. Chronic hyperinsulinemia plays a central role in causing IR in cirrhosis^[41]^. HD differs from T2D, with less microangiopathy but more cirrhotic complications and increased mortality^[38]^. Hepatitis C, alcoholic cirrhosis, and NAFLD are frequently associated with HD^[38,42]^. Management of HD is complex due to potential hepatotoxicity of oral hypoglycemic drugs and the risk of certain anti-diabetic agents promoting hepatocarcinogenesis^[38,42]^.

## Clinical correlations and outcomes

### Impact of DM on cirrhotic patients

Chronic liver diseases, particularly cirrhosis, are strongly associated with glucose intolerance and HD. About 80% of cirrhotic patients exhibit glucose intolerance, with 20–30% developing diabetes^[41]^. HD prevalence in cirrhosis ranges from 30 to 60%^[38]^. The pathophysiology involves IR in muscle, liver, and adipose tissues, along with hyperinsulinemia and impaired pancreatic β-cell response^[38,41]^. Chronic hyperinsulinemia plays a central role in causing IR in cirrhosis^[41]^. HD differs from T2D, with less microangiopathy but more cirrhotic complications and increased mortality^[38]^. Hepatitis C, alcoholic cirrhosis, and NAFLD are frequently associated with HD^[38,42]^. Management of HD is complex due to potential hepatotoxicity of oral hypoglycemic drugs and the risk of certain anti-diabetic agents promoting hepatocarcinogenesis^[38,42]^.

### Diabetic patients with liver dysfunction

NAFLD is highly prevalent among T2D patients, affecting over half of this population^[43,44]^. NAFLD encompasses a spectrum from simple steatosis to NASH, fibrosis, and cirrhosis. Diabetes is an independent risk factor for advanced liver disease progression and hepatocellular carcinoma^[43,45]^. The high prevalence of NAFLD in diabetic patients is partly due to IR, which plays a crucial role in its development^[43]^. Despite its significance, NAFLD remains underdiagnosed and underrecognized in primary care settings^[46]^. Diagnosis challenges are similar for diabetic and non-diabetic patients, involving imaging, serological methods, and histopathological evaluation^[45]^. The interplay between diabetes and NAFLD involves mutual pathogenetic mechanisms, with genetic and environmental factors potentially accelerating NAFLD progression in diabetic patients^[45]^. Clinical corelates and outcomes are summarized in Table 2.

**Table 2:** Clinical correlations and outcomes in diabetes mellitus (DM) and cirrhosis

Clinical correlation	Description
Hepatogenous diabetes (HD) in cirrhotic patients	Glucose intolerance is seen in ~80% of cirrhotic patients, with 20–30% developing diabetes; HD occurs in 30–60% of cirrhosis cases. It features insulin resistance, hyperinsulinemia, and altered β-cell function. HD has fewer microvascular complications than type 2 DM but is associated with worse cirrhotic outcomes and higher mortality.
Etiological associations	Hepatitis C, alcoholic cirrhosis, and NAFLD are common underlying causes.
Management challenges	Use of oral hypoglycemics is complicated due to hepatotoxicity and potential for hepatocarcinogenesis with certain antidiabetic drugs.
NAFLD in diabetic patients	Over 50% of type 2 diabetics have NAFLD, ranging from steatosis to cirrhosis. Diabetes accelerates disease progression and increases HCC risk.
Pathogenesis and risk factors	Insulin resistance, shared genetic and environmental contributors.
Diagnostic difficulties	Underdiagnosed in primary care; evaluation involves imaging, serological markers, and histology, similar to non-diabetic populations.

HCC, hepatocellular carcinoma; NAFLD, non-alcoholic fatty liver disease.

## Implications for screening and diagnosis

Recent studies highlight the importance of screening for NAFLD and advanced fibrosis in patients with T2DM. Incorporating liver fibrosis assessment into routine diabetic care significantly improves identification of advanced liver disease^[47]^. A two-tier screening approach using FIB-4 followed by transient elastography is recommended for efficient risk stratification^[48]^. Research indicates that moderate-to-advanced fibrosis affects approximately 15% of T2DM patients, supporting the need for systematic screening^[49]^. While liver enzymes alone are insufficient for initial screening, combining them with imaging and plasma diagnostic panels enhances performance^[50]^. Applying these screening strategies to the U.S. population suggests that advanced liver fibrosis can be excluded in 50–67% of T2DM patients using non-invasive methods, potentially reducing the need for liver biopsies^[50]^.

Given the bidirectional risk between T2DM and cirrhosis, systematic screening for liver disease in diabetic patients is essential. Evidence supports integrating FIB-4 scoring with transient elastography into *annual diabetes reviews* to identify advanced fibrosis early. Conversely, cirrhotic patients should be routinely evaluated for glucose intolerance and HD using OGTT or HbA1c, even in the absence of hyperglycemic symptoms. A multidisciplinary approach is critical; *endocrinologists* can optimize glycemic control and minimize hepatotoxic medication use, *hepatologists* can tailor fibrosis surveillance and manage portal hypertension risks, *dietitians & exercise physiologists* can guide weight loss programs targeting ≥10% reduction for histologic benefit, and *primary care physicians* can coordinate ongoing monitoring, ensuring patients remain in care loops for both conditions.

## Therapeutic considerations

### Glycemic control and liver outcomes

Glycemic control plays a crucial role in managing NAFLD in patients with T2DM. Tight glycemic control has been shown to slow liver disease progression and improve histological outcomes in NAFLD (Bloomgarden, 2017; García-Compeán et al., 2022). Studies have demonstrated that improvements in HbA1c levels correlate with reductions in liver fat and fibrosis (Bloomgarden, 2017)^[59]^. Weight loss of >10% and good glycemic control are associated with histological improvements in NAFLD (García-Compeán et al., 2022)^[60]^. The co-existence of NAFLD and T2DM increases the risk of complications in both conditions, emphasizing the importance of managing both diseases simultaneously (Hazlehurst et al., 2016)^[61]^. While therapeutic options for NAFLD are limited, achieving good glycemic control and optimizing weight loss are pivotal in restricting disease progression (Hazlehurst et al., 2016)^[61]^. Recent research suggests that the degree of glycemic control may predict the severity of hepatocyte ballooning and hepatic fibrosis in NAFLD (Alexopoulos et al., 2021)^[62]^.

### Antidiabetic drugs with hepatic benefits

Recent studies highlight the potential hepatoprotective effects of antidiabetic drugs. Metformin, a first-line treatment for T2D, has shown promise in improving hepatic steatosis and suppressing liver inflammation through AMPK-dependent and independent pathways^[51,52]^. Sodium-glucose cotransporter-2 inhibitors demonstrate extra-glycemic benefits and protective effects on the liver, offering potential for treating various hepatic disorders^[53]^. Glucagon-like peptide-1 (GLP-1) receptor agonists have also garnered interest for their impact on NAFLD and NASH^[54]^. These newer antidiabetic drugs show potential in addressing the increasing prevalence of NAFLD/NASH, which is closely associated with T2D. Their ability to improve liver function and histology suggests significant benefits for human health, particularly in patients with chronic liver diseases^[51,54]^.

### Liver-directed therapies and effect on glucose metabolism

Liver-directed therapies have shown promising effects on glucose metabolism in various liver conditions. Ischemic therapy and glucose metabolism interference have been explored for treating liver malignancies, with potential benefits in reducing tumor growth^[55]^. In metastatic insulinoma, liver-directed therapies like chemoembolization and radioembolization effectively manage intractable hypoglycemia, increasing blood glucose levels and reducing symptomatic episodes^[56]^. Direct-acting antiviral treatment for hepatitis C has been associated with improved glucose metabolism, including reduced fasting glucose levels and rates of impaired fasting plasma glucose, particularly in patients without liver cirrhosis^[57]^. Various drugs targeting liver metabolism are being investigated for NAFLD and steatohepatitis, including nuclear hormone receptor agonists, incretins, and sodium-glucose cotransporter inhibitors, which may improve both diabetic hyperglycemia and fatty liver disease^[58]^. All therapeutic considerations are summarized in Table 3.

**Table 3:** Therapeutic considerations in diabetes and liver disease

Therapeutic focus	Description
Glycemic control in NAFLD	Tight glycemic control improves histological liver outcomes and slows NAFLD progression in type 2 diabetes patients. HbA1c reduction correlates with less liver fat and fibrosis.
Weight loss as adjunct	Weight loss >10% is associated with significant histological improvement in NAFLD; co-management of T2DM and NAFLD is crucial due to shared risk factors.
Metformin	Enhances hepatic steatosis resolution and reduces liver inflammation via AMPK-dependent and independent mechanisms.
SGLT2 inhibitors	Offer liver protection and extra-glycemic benefits; potential use in NAFLD and other liver disorders.
GLP-1 receptor agonists	Shown to improve liver function and histology in NAFLD/NASH; emerging therapeutic option.
Liver-directed therapies	Techniques like chemoembolization and radioembolization improve glucose regulation in liver tumors and insulinomas; may also benefit metabolic dysfunction.
Direct-acting antivirals for HCV	Improve fasting glucose and insulin sensitivity post-treatment, especially in non-cirrhotic patients.
Emerging liver-targeted drugs	Includes nuclear hormone receptor agonists, incretins, and SGLT inhibitors – aimed at treating both NAFLD/NASH and diabetic hyperglycemia.

GLP-1, glucagon-like peptide-1; NAFLD, non-alcoholic fatty liver disease; NASH, non-alcoholic steatohepatitis; T2DM, type 2 diabetes mellitus; SGLT2, sodium-glucose cotransporter-2; HCV, hepatitis C virus; AMPK, AMP-activated protein kinase; HbA1c, glycated hemoglobin.

## Future directions and research needs

MASLD and T2DM frequently coexist and accelerate each other’s progression. This overlap is largely driven by shared mechanisms such as IR, chronic inflammation, and hepatic lipid toxicity, underscoring the need for integrated strategies in diagnosis and treatment. To move beyond general calls for collaboration, we recommend several concrete actions. First, conduct randomized controlled trials on agents such as pioglitazone, GLP-1 receptor agonists, and emerging therapies (e.g., dual incretin agonists, farnesoid X receptor (FXR) agonists) to assess their simultaneous effects on glycemic control and hepatic histology. Second, develop and validate combined NAFLD–T2DM risk calculators that incorporate metabolic, hepatic, and cardiovascular parameters to enable earlier identification of high-risk patients. Third, identify and validate circulating or imaging-based biomarkers capable of predicting disease progression in both MASLD and T2DM, allowing for targeted early interventions. Fourth, test clinical care models in which hepatologists and endocrinologists collaborate directly with nutritionists and other allied health professionals to improve adherence, continuity of care, and patient outcomes. Finally, investigate whether aggressive early management of one condition (e.g., intensive glycemic control) can alter the trajectory of the other (e.g., regression of hepatic fibrosis). Implementing these measures will require healthcare systems to promote closer integration among liver specialists, diabetes teams, and primary care providers. By addressing metabolic, hepatic, and cardiovascular risks collectively rather than in isolation, future research can help deliver more effective interventions and ultimately reduce the burden of severe illness and mortality associated with both MASLD and T2DM.

## Conclusion

The growing global prevalence of T2DM and MASLD underscores an urgent need to address their deeply interconnected pathophysiology. This paper has outlined the shared mechanistic pathways, including IR, lipid toxicity, chronic inflammation, microvascular dysfunction, and the role of advanced glycation end-products, that link T2DM and liver cirrhosis. Evidence from epidemiological studies and clinical observations further supports a bidirectional relationship, where each condition exacerbates the other’s severity and risk of complications, including hepatocellular carcinoma and cardiovascular morbidity.

Clinical implications are far-reaching. Patients with diabetes often harbor unrecognized liver dysfunction, while those with cirrhosis are at elevated risk for developing HD. This interconnected disease burden calls for integrated screening protocols, early detection strategies, and personalized therapeutic approaches that consider both metabolic and hepatic health. Non-invasive diagnostic tools, combined with a multidisciplinary care model, can significantly improve outcomes for this high-risk population.

Ultimately, understanding the interplay between diabetes and chronic liver disease is essential not only for guiding clinical management but also for shaping public health policies aimed at mitigating the long-term consequences of these increasingly prevalent disorders. Future research should prioritize longitudinal studies, innovative biomarker development, and targeted therapeutics to better prevent and manage the dual burden of T2DM and liver cirrhosis.

## Data Availability

Not applicable for this review.
